# The Dilemma and Wisdom in Translating *p* Values: A Collaborative Approach to Strengthening Scientific Validity

**DOI:** 10.1155/bmri/6703756

**Published:** 2025-03-31

**Authors:** Mohamad Adam Bujang

**Affiliations:** Clinical Research Centre, National Institutes of Health, Sarawak General Hospital, Ministry of Health Malaysia, Kuching, Sarawak, Malaysia

**Keywords:** inferential analyses, *p* value, significant, statistical analysis

## Abstract

The *p* value remains a widely used statistical tool for assessing the significance of research findings, yet its interpretation and application often provoke debate. While the *p* value provides a measure of evidence against the null hypothesis, it does not convey essential information about the strength of an effect or its statistical precision. Therefore, this paper critically examines the dilemma of translating *p* values into meaningful scientific inferences. Using illustrative examples, we demonstrate how the misapplication of *p* values can misguide policy, clinical practice, and scientific discourse. To address these challenges, this article proposes a collaborative approach involving researchers, journal reviewers, and the scholarly audience. This includes a comprehensive checklist to promote clear documentation, critical evaluation, and a more nuanced interpretation of statistical evidence. By fostering a nuanced understanding of statistical evidence, this initiative seeks to advance scientific validity and ensure that research findings contribute meaningfully to societal progress.

## 1. Introduction

In hypothesis testing, the *p* value is a statistical measure that quantifies the strength of evidence against the null hypothesis. Conventionally, a *p* value less than 0.05 is considered statistically significant, leading researchers to infer that the observed effect is unlikely to be due to chance alone. This threshold has been widely adopted in scientific research to assess the strength of evidence against the null hypothesis. However, statistical significance alone does not prove or disprove the validity of study findings. It simply shows whether the data go beyond common *p* value thresholds (e.g., 0.05 or 0.001) and indicates how well the data fit with the null hypothesis, assuming it is true, which is a hypothetical assumption [1–3].

Despite their widespread use in scientific research, the interpretation of *p* values has long been debated among statisticians, researchers, and policymakers [4–7]. The primary role of inferential analysis is to draw conclusions about a population based on sample data. To achieve this, it uses statistical methods to estimate parameters and test hypotheses with a known degree of uncertainty. However, because these conclusions rely on sample data, the true population parameter remains unknown. Statistical methods can provide estimates, but some level of uncertainty always persists unless the statistics have been validated [8]. While *p* values are not mathematically flawed, their value lies in helping assess the likelihood of data extreme enough to challenge the null hypothesis. However, they should not be relied upon alone for practical significance or causality.

Statistical significance testing relies on arbitrary thresholds, such as 0.05 or 0.01, to categorize results as either “significant” or “not significant.” This dichotomous approach has been widely criticized as it fosters binary thinking, potentially overemphasizing trivial findings while dismissing meaningful trends. In contrast, the *p* value is a continuous metric that quantifies the compatibility of the observed data with the null hypothesis. Thus, a major limitation of the *p* value lies in its nature as a single numerical summary. It provides no information about the magnitude or precision of an observed effect, offering only a test of the data's compatibility with the null hypothesis [1–3].

To strengthen the validity and reproducibility of research findings, researchers are encouraged to present *p* values alongside confidence intervals and effect sizes, as this provides a more comprehensive framework for evaluating results, highlighting both statistical and practical significance. This integrated approach helps mitigate the risks of misinterpretation and fosters a deeper understanding of scientific evidence in both theoretical and applied contexts [9–11].

Therefore, relying on *p* values to draw conclusions is dangerous. A *p* value is merely one statistical indicator, highly dependent on sample data, and does not provide direct evidence of the truth or magnitude of an effect. It is essential to recognize that a *p* value is a probabilistic outcome derived from the framework of hypothesis testing and it does not offer a definitive measure of an effect's importance or real-world relevance. Thus, *p* values must be interpreted within a broader context. To properly understand their significance, we must first understand the origin of *p* value by start discussing between descriptive and inferential statistics. Descriptive statistics summarize data and present characteristics of the sample without making any claims about the population. Inferential statistics, on the other hand, are aimed at drawing conclusions about a population based on sample data through techniques such as hypothesis testing and estimation [12, 13].

For studies involving sample-based data, descriptive statistics alone are insufficient to make robust inferences about the population. This is where inferential analysis becomes critical. *p* values offer one piece of evidence for assessing whether the findings in the sample can be generalized to the population. A *p* value less than 0.05 is commonly interpreted as suggesting that the observed effect in the sample likely reflects a real effect in the population. However, this interpretation is not absolute since a small *p* value does not equate to definitive proof of an effect, nor does it guarantee that the effect is substantial or practically meaningful [12–14].

## 2. Misinterpretation of *p* Values in Observational Studies to Claim for an Effect

Misuse of *p* values is particularly common in observational studies, where they are often used to make claim of relationships between variables, such as associations, correlations, or differences [15, 16]. In such cases, researchers may misinterpret *p* values if they overlook crucial considerations such as effect sizes, confidence intervals, or confounding factors. Ignoring these elements can lead to spurious conclusions that overstate the strength or validity of findings [13, 14].

Consider a study examining the correlation between knowledge scores and practice scores in a specific field and condition. Suppose the results show a correlation coefficient of 0.2, with a 95% confidence interval of (0.1, 0.3) and a *p* value less than 0.05. While this statistical analysis suggests a weak but statistically significant correlation, it is important for researchers to avoid overstating the relationship between knowledge and practice. The conclusion that “there is a correlation between knowledge and practice” is technically correct based on statistical significance, but it does not fully reflect the practical significance or the strength of the correlation. In interpreting such results, researchers should consider both the magnitude of the correlation coefficient and the *p* value, as this combined approach provides a more nuanced and comprehensive understanding of the relationship between the variables.

The problem worsens when more and more scholars cite the study's conclusion without scrutinizing the details of the results, particularly the small effect size and confidence interval. Over time, this can lead to the widespread acceptance of the notion that knowledge and practice pertaining to the previous example are correlated in that specific field and condition. Policymakers, practitioners, or educators might then apply this conclusion to real-world practice, expecting knowledge to meaningfully impact practice outcomes.

However, because the correlation coefficient is so small (effect size of 0.2), it indicates a weak relationship [17, 18]. In practical terms, such a low correlation means that increasing knowledge might have a minimal effect on practice. If these findings are applied in real-world scenarios, practitioners might be surprised to find that improvements in knowledge do not lead to significant improvements in productivity or performance. The statistics themselves did not “cheat” or mislead. Rather, the issue arises from misinterpretation because of failing to recognize that statistical significance does not imply practical significance.

On the other hand, the real story behind the scene is that the researchers have, first, gathered sufficient evidence to infer this result to the target population and, second, determined that the correlation between knowledge and practice is low. Assuming that the scales used to measure both knowledge and practice are valid and reliable, there are several recommendations that can be drawn from the results, depending on the motive of the study. If the primary goal of the study is simply to measure the correlation between the two variables, the appropriate conclusion would be that there is a low correlation. The researchers should then clearly communicate that, although statistically significant, the relationship between knowledge and practice is weak. In such cases, further research might focus on identifying other factors that could explain variability in practice scores, beyond just knowledge.

Hence, if the study's intent was to demonstrate a strong correlation between knowledge and practice, then the low correlation found here suggests a gap. The researchers would need to reassess their approach. This could involve exploring interventions to strengthen both knowledge and practice. In this case, the findings would prompt a need for additional strategies or interventions aimed at improving both knowledge and practice to achieve the desired high correlation. In either scenario, the interpretation of the results should be aligned with the objectives of the study. If the goal is purely to gain information, the low correlation is a valuable finding on its own. However, if the goal is prescriptive or action-oriented, such as finding ways to enhance practice through knowledge, the low correlation indicates that additional efforts are needed to bridge that gap.

## 3. Misinterpretation of *p* Values to Determine Associated or Risk Factors of an Outcome

Consider a study examining the association between high meat consumption and the risk of developing colorectal cancer. Say the results show a relative risk of 1.3, with a 95% confidence interval of 1.10–1.50 and a *p* value less than 0.05. While the statistical analysis indicates an association between high meat consumption and colorectal cancer, if researchers are not careful, they may overstate the strength of this association. In this context, the conclusion that high meat consumption is associated with an increased risk of colorectal cancer may be technically correct in terms of statistical significance, but it does not fully capture the magnitude or practical relevance of the association.

The problem intensifies when many scholars cite a study's conclusions without carefully scrutinizing the details, particularly the effect size and confidence intervals. Over time, this can foster the perception that high meat consumption is a major risk factor for colorectal cancer. As a result, public health campaigns or dietary guidelines may prioritize cutting down on meat as a key cancer prevention strategy. However, with a relative risk of just 1.3, the effect size is fairly modest [17]. In practical terms, while high meat consumption may slightly raise the risk of colorectal cancer, it is likely far from being the sole or most critical factor. Healthcare professionals could find themselves puzzled when advising patients to eat less meat yields only marginal reductions in cancer cases.

On the other note, consider a study investigating the risk factors linked to a particular outcome. The results show odds ratios (ORs) and *p* values as follows: gender with OR = 1.2, *p* < 0.05; age group with OR = 3.0, *p* < 0.05; highest education level with OR = 1.2, *p* < 0.05; and type of treatment with OR = 3.5, *p* < 0.05. Traditionally, some researchers might declare all four factors as significant risk factors for the outcome. However, in reality, gender and education level have a minimal impact. Their statistical significance could be driven by the large sample size, as their effect sizes are small [12, 17].

If the goal of the study is simply to identify whether these variables are associated with the outcome, regardless of effect size, it is valid to conclude that all four are significant [19]. But, if the objective is to pinpoint the actual risk factors, the conclusion should be more nuanced: There are four variables significantly associated with the outcome. However, only two variables such as age group (OR = 3.0, *p* < 0.05) and type of treatment (OR = 3.5, *p* < 0.05) show substantial effect sizes and can be considered genuine risk factors. This highlights the importance of interpreting multivariate regression models with caution, especially in studies with very large sample sizes, where statistical significance does not always equate to meaningful impact.

## 4. Misinterpretation of *p* Values in Experimental Studies

In experimental studies, *p* values are frequently used to evaluate the effectiveness of an intervention or treatment [20, 21]. This is where significant misuse has occurred. Although *p* values have been conventionally employed for making inferences, in recent decades they have been increasingly misapplied as a binary indicator to assert that a hypothesis is definitively true or false. In reality, a *p* value alone does not provide certainty, nor does it validate the correctness of a hypothesis. Statistically significant results can be misleading, particularly when derived from studies with small sample sizes, poor study design, or data-driven testing [22, 23].

Consider a randomized controlled trial examining the effect of a new medication on reducing symptoms of anxiety. The results indicate a mean difference of 2.5 points on a standardized anxiety scale of 100 units, with a 95% confidence interval of 0.5 to 4.5 and a *p* value less than 0.05. While the result suggests that the medication has an effect on reducing anxiety symptoms, if researchers are not careful, they may overstate the efficacy of the treatment. In this context, the conclusion that “the new medication effectively reduces anxiety symptoms” may be technically correct in terms of statistical significance, but it does not fully account for the clinical relevance of the findings.

The issue becomes more pronounced when other scholars cite the study's conclusion without closely examining the details of the results, particularly the effect size and confidence interval. Over time, this could lead to the widespread acceptance of the medication as a breakthrough treatment for anxiety. Clinicians might then prescribe the medication based on the assumption that it will yield significant improvements for all patients. However, a mean difference of 2.5 points, while statistically significant, may not represent a clinically meaningful change for individuals experiencing anxiety. If these findings are applied in real-world clinical settings, healthcare practitioners might be surprised when patients do not experience the expected level of symptom relief.

Therefore, researchers must approach the interpretation of RCT findings with caution. If the goal is to determine whether the medication has a statistically significant effect on anxiety symptoms, the results should make it clear that while an effect is present, its clinical relevance may still be uncertain. However, if the aim is to inform treatment guidelines, the modest effect size suggests that healthcare providers should adopt a holistic approach to treating anxiety such as incorporating psychotherapy, lifestyle modifications, and other therapies alongside medication. Otherwise, policymakers might consider discontinuing or banning the medication altogether.

## 5. Clarity of Relationship Between Study Design and *p* Value

It was not the author's intention to conflate study design (i.e., observational or experimental studies) with the meaning or limitations of *p* values. The purpose was to provide different examples of misconceptions in the interpretation of *p* values across various study designs and their respective objectives. This approach was intended to offer clear, context-specific examples to illustrate how such misinterpretations can arise, with the goal of helping readers avoid these bad practices and improve the quality of statistical analysis in research. This study emphasizes the importance of appropriately assessing the *p* value and ensuring the correct and valid interpretation of any result derived from inferential analysis regardless of the study design. This is because understanding the limitations and context of statistical measures is essential for drawing meaningful conclusions.

## 6. Recommended Solutions

In the realm of scientific research, *p* values play a crucial role in assessing the statistical significance of study results. However, misinterpretations of *p* values can lead to flawed conclusions and misguided decision-making. To mitigate these risks, it is essential for researchers, journal reviewers, and the scholarly audience to understand their respective roles in promoting a more nuanced interpretation of *p* values [24, 25]. By implementing recommendations tailored to each group, the scientific community can foster greater clarity and accuracy in the communication of research findings.

### 6.1. The Role of Researchers

Researchers are at the forefront of the fight against misinterpretation of *p* values. One of their primary responsibilities is to provide transparent reporting of statistical methods. This includes detailing not only *p* values but also effect sizes and respective confidence interval (when it is applicable). By offering a comprehensive view of their analyses, researchers can help readers grasp the practical implications of their findings. Furthermore, it is imperative for researchers to emphasize the context surrounding their results. By clearly discussing the clinical or scientific significance of their findings alongside statistical significance, they can ensure that readers do not misconstrue statistical results as definitive conclusions. In addition, researchers should advocate for greater statistical literacy within their fields. This can be accomplished by incorporating explanations of *p* values and their proper interpretation in publications. By demystifying *p* values, researchers can foster a more nuanced understanding among their peers and readers, reducing the likelihood of misinterpretation.

### 6.2. The Role of Reviewers

Journal reviewers also play a vital role in minimizing the misinterpretation of *p* values. They must rigorously evaluate the statistical analyses presented in submitted manuscripts, ensuring that authors provide a balanced view that includes *p* values, effect sizes, and confidence intervals. Reviewers should encourage authors to discuss the practical significance of their findings, helping to contextualize statistical results within real-world applications. Moreover, reviewers can advocate for journals to adopt guidelines that require authors to include both *p* values and effect sizes in their manuscripts. Such standardization in reporting practices will facilitate better interpretation by the audience and promote a more comprehensive understanding of research findings.

Moreover, scientific journals should emphasize the importance of reviewers addressing specific cautions regarding the interpretation of *p* values in the articles they review. Reviewers play a critical role in the peer-review process, acting as gatekeepers of quality and rigor in scientific publishing. Therefore, it is essential for journals to provide clear guidelines encouraging reviewers to scrutinize not only the statistical analyses presented in the manuscripts but also the interpretations made by the authors concerning *p* values. Reviewers should be prompted to evaluate whether the authors have adequately discussed the limitations of *p* values and the context in which they are interpreted. This includes assessing whether the authors have provided sufficient information on effect sizes or/and confidence intervals and the practical significance of their findings. By encouraging reviewers to highlight these aspects, journals can help ensure that researchers do not rely solely on *p* values to draw broad conclusions about their findings.

Furthermore, journals can establish criteria for reviewers to assess the clarity and transparency of statistical reporting. This could involve asking reviewers to comment on whether the authors have appropriately contextualized their *p* values, particularly in relation to the study's objectives and the broader implications of the research. This emphasis on rigorous review will encourage authors to present their results in a more balanced manner, reducing the risk of misinterpretation by the readership. By implementing these recommendations, scientific journals can significantly reduce the risk of misinterpretation of *p* values in published research, ultimately leading to more reliable conclusions and applications in real-world contexts.

Indeed, the role of the reviewer is crucial in acting as a key control point for preventing the misinterpretation of *p* values between authors and the wider audience, including scholars, policymakers, and practitioners. As gatekeepers in the peer-review process, reviewers are responsible for ensuring that statistical analyses, such as *p* values, are properly applied, interpreted, and reported. Their careful scrutiny can prevent the propagation of misleading conclusions and promote a more rigorous understanding of research findings.

### 6.3. The Role of Scholars and the Audience

Finally, scholars and the broader audience must engage critically with research. This involves evaluating not just *p* values but also the accompanying effect sizes, confidence intervals, study design, sample size, and potential biases present in the research. Scholars should foster discussions around the broader context of research findings, recognizing that statistical significance does not equate to practical significance. Continuing education on statistics is crucial for scholars and the audience alike. By seeking opportunities for professional development such as workshops, seminars, or online courses, they can enhance their understanding of statistical concepts, including the limitations and proper interpretation of *p* values. This commitment to education will empower scholars to engage with research more critically and responsibly.

## 7. Summary

The *p* value is a useful tool in statistical analysis if and only if it is well interpreted and discussed. The *p* value is often misinterpreted and overemphasized in scientific research, which can lead to flawed conclusions and misguided decisions. Such misinterpretation can have serious consequences for research. Despite its limitations, the *p* value is widely used and recognized globally, making it difficult to eliminate from statistical practices. Therefore, to mitigate these issues, it is crucial for all stakeholders which include researchers, journal reviewers, and the scholarly audience to collaborate and actively participate in promoting accurate interpretation of *p* values. These efforts are crucial to strengthening the scientific validity of research discoveries, ensuring that findings are accurately interpreted and applied. To end the discussion, Table 1 presents a recommended checklist that highlights the responsibilities of each group in ensuring proper use and interpretation of *p* values in scientific research. By promoting careful and responsible use of statistical tools like *p* values, we can reduce the likelihood of misleading conclusions and, ultimately, foster real enhancements in various fields of knowledge. This will translate into better decision-making, improved policies, and tangible benefits in real-world applications that positively impact lives.

## Figures and Tables

**Table 1:** Checklist for researchers, journals (including reviewers), and audience to avoid misinterpretation of *p* value.

**Checklist**
*For researchers:*
1. Clearly specify the types of statistical tests employed in the study. Provide an explanation of what *p* values represent within the context of the research, including details about the statistical analyses conducted. The authors should include this information in the Methods section or, alternatively, in the footnotes of any tables or figures that involve inferential analysis.
2. Report effect sizes or other relevant statistical indicators. Include effect sizes or other appropriate statistical indicators alongside *p* values to provide context regarding the strength and practical significance of the results. Other relevant indicators may include mean differences and proportion differences whenever is relevant and appropriate.
3. Provide confidence intervals (when is relevant and appropriate, i.e., to determine risk factors especially in a multivariate model, interventional studies). Present 95% confidence intervals for estimates to offer insight into the precision of your findings whenever is applicable.
4. Discuss scientific relevant and practical significance of the finding(s). Discuss the magnitude of correlation, association, difference, etc. based on effect sizes of other relevant indicators such as mean differences and proportion differences.
5. Acknowledge limitations in the inferential analysis. Be cautious when interpreting statistical significance if a very large sample size was used in the study, as it could lead to excessively small *p* values.

*For journals and reviewers:*
1. Evaluate statistical technique(s) used in the study: Scrutinize the statistical methods used and whether they align with the study's objectives.
2. Assess clarity of reporting: Check if the authors clearly report *p* values, effect sizes or other relevant statistical indicators and confidence intervals (whenever is relevant and appropriate). Ensure these metrics are presented in a comprehensible manner.
3. Encourage contextualization: Ensure authors to discuss the implications of their statistical results within the context of their research and field.
4. Critique interpretation of results: Provide feedback on whether the authors' conclusions based on statistical results are justified given the data and effect sizes (or other relevant statistical indicators, i.e., mean differences) reported.
5. Publish transparent methods: The authors should provide a detailed explanation of the methodology, including the study design, sample characteristics, instruments used, data collection methods, and statistical analysis techniques. The reviewers should assess the appropriateness of the research design in relation to the statistical analyses applied in the study.
6. Report limitation of study: The authors need to address the possibility that the reported *p* values may be small due to the inclusion of a very large sample size in the study. Specifically, they should consider discussing whether the sample size increased the statistical power of the study, potentially leading to statistically significant results even for small or trivial effects.

*For scholars and the audience:*
1. Critically evaluate research: Look beyond *p* values and assess the entire statistical report, including effect sizes, other relevant statistical indicators (i.e., mean differences), and confidence intervals (if available).
2. Understand the limitations: Recognize that very small *p* values do not usually indicate practical significance and can be influenced by very large sample size.
3. Consider the research design: Evaluate the robustness of the study design and methods used before accepting conclusions based solely on *p* values.
4. Encourage to conduct further literature review: Look and review on other relevant studies to compare or validate findings and provide a clearer understanding and the credibility of scientific articles.
5. Promote foster communication with statistician: Discuss with statisticians to enhance the understanding on the article.

## Data Availability

Data sharing is not applicable to this article as no new data were created or analyzed in this study.

## References

[1] Held L., Sabanés Bové D. (2014). *Applied Statistical Inference–Likelihood and Bayes*.

[2] Casella G., Berger R. L. (2002). *Statistical Inference*.

[3] Rothman K. J. (2021). The origin of modern epidemiology, the book. *Journal of Epidemiology*.

[4] Rothman K. J. (1978). A show of confidence. *New England Journal of Medicine*.

[5] Rothman K. J. (2016). Disengaging from statistical significance. *European Journal of Epidemiology*.

[6] Amrhein V., Greenland S., McShane B. (2019). Scientists rise up against statistical significance. *Nature*.

[7] Berselli N., Filippini T., Adani G., Vinceti M. (2021). Dismissing the use of *P*-values and statistical significance testing in scientific research: new methodological perspectives in toxicology and risk assessment. *Toxicological Risk Assessment and Multi-System Health Impacts from Exposure*.

[8] Bujang M. A., Sa’at N., Joys A. R., Ali M. M. (2015). An audit of the statistics and the comparison with the parameter in the population. *AIP Conference Proceedings*.

[9] Schatz P., Jay K. A., McComb J., McLaughlin J. R. (2005). Misuse of statistical tests in publications. *Archives of Clinical Neuropsychology*.

[10] Fidler F., Burgman M. A., Cumming G., Buttrose R., Thomason N. (2006). Impact of criticism of null-hypothesis significance testing on statistical reporting practices in conservation biology. *Conservation Biology*.

[11] Hoekstra R., Finch S., Kiers H. A. L., Johnson A. (2006). Probability as certainty: dichotomous thinking and the misuse of *p* values. *Psychonomic Bulletin & Review*.

[12] Bujang M. A. (2021). A step-by-step process on sample size determination for medical research. *Malaysian Journal of Medical Sciences: MJMS*.

[13] Wasserstein R. L., Lazar N. A. (2016). The ASA statement on *p*-values: context, process, and purpose. *The American Statistician*.

[14] Greenland S., Senn S. J., Rothman K. J. (2016). Statistical tests, *P* values, confidence intervals, and power: a guide to misinterpretations. *European Journal of Epidemiology*.

[15] Ludwig D. A. (2005). Use and misuse of p-values in designed and observational studies: guide for researchers and reviewers. *Aviation, Space, and Environmental Medicine*.

[16] Jepsen P., Johnsen S. P., Gillman M. W., Sørensen H. T. (2004). Interpretation of observational studies. *Heart*.

[17] Cohen J. (1992). A power primer. *Psychological Bulletin*.

[18] Bujang M. A. (2024). An elaboration on sample size determination for correlations based on effect sizes and confidence interval width: a guide for researchers. *Restorative Dentistry & Endodontics*.

[19] Bujang M. A., Sa'at N., Sidik T. M. I. T. A. B., Joo L. C. (2018). Sample size guidelines for logistic regression from observational studies with large population: emphasis on the accuracy between statistics and parameters based on real life clinical data. *Malaysian Journal of Medical Sciences: MJMS*.

[20] Butcher N. J., Monsour A., Mew E. J. (2022). Guidelines for reporting outcomes in trial reports: the CONSORT-outcomes 2022 extension. *Jama*.

[21] Yu L. M., Chan A. W., Hopewell S., Deeks J. J., Altman D. G. (2010). Reporting on covariate adjustment in randomised controlled trials before and after revision of the 2001 CONSORT statement: a literature review. *Trials*.

[22] McShane B. B., Gal D., Gelman A., Robert C., Tackett J. L. (2019). Abandon statistical significance. *American Statistician*.

[23] Colquhoun D. (2014). An investigation of the false discovery rate and the misinterpretation of *p*-values. *Royal Society Open Science*.

[24] Lederer D. J., Bell S. C., Branson R. D. (2019). Control of confounding and reporting of results in causal inference studies. Guidance for authors from editors of respiratory, sleep, and critical care journals. *Annals of the American Thoracic Society*.

[25] Savitz D. A., Wise L. A., Bond J. C. (2024). Responding to reviewers and editors about statistical significance testing. *Annals of Internal Medicine*.

